# There’s been a Flaw in Our Thinking

**DOI:** 10.3389/fimmu.2014.00540

**Published:** 2014-10-31

**Authors:** Clark L. Anderson

**Affiliations:** ^1^Division of Immunology, Department of Internal Medicine, The Ohio State University, Columbus, OH, USA

**Keywords:** FcRn, IgG, transport, albumin, turnover rate

Rogers Brambell (1901–1970), the father of the field of FcRn biology, was by all accounts a scientist of great imagination and insight, one whom we would look to, were he available, for opinions on our current scientific direction. Were we to ask him, in a moment of fantasy, to review recent progress in his field, we think he would say that we have gone astray, that we have become confused about what he thought was a critical issue. Specifically, we have ignored the important point of where in the cell FcRn expresses its specificity for ligand, either on or in the cell; and rather than deal directly with that basic issue, we have side-stepped the question, leaving it unanswered, and in fact have implied that it is not a critical question.

His last comment on the issue, in his 1970 tome, published the year he died, was that specificity of the receptor for ligand in tissues like the yolk sac was determined intracellularly in what we now know to be acidic vesicles of the pinocytic trafficking pathway. Thus receptor-bound ligand destined for transcellular transport was separated effectively from free ligand destined for lysosomal degradation. To others, however, the situation seemed more complex, and alternate views were expressed. In the gut of the neonatal rat or mouse, the receptor was found expressed on the epithelial surface where under the influence of the low pH of gut contents it bound with high affinity to the IgG in maternal milk, and both the receptor and its bound ligand were endocytosed, the ligand ultimately reaching its destination, the fetal circulation. Receptor specificity for ligand, thus, was conferred at the cell surface.

For receptor–ligand specificity to be dictated at two different sites depending on the nature of the tissue seemed far-fetched to some, and additional observations were brought to bear. The low pH of gut contents was questioned. Gut pH had been measured only once, with litmus paper, and the observation was never repeated. As well, the relevance of surface-expressed receptor was questioned. It became apparent that only a fraction of total cell receptor, probably <1%, is found on the surface of the cell. This low level of surface expression is likely a vestige of the exocytosis step of IgG transport and not an essential component of the ligand-uptake pathway. Further, an experiment expressly designed to evaluate the effect of gut pH on IgG transfer to the neonate indicated that acidic pH was not necessary; non-specific uptake of ligand into the cell was adequate (1).

Despite underlying doubts about the physiologic relevance of surface-displayed receptor, it proved virtually impossible to perform *in vitro* studies of IgG uptake by cultured cells unless the medium was made acidic. Uptake at physiologic pH was near nil. Thus, workers proceeded to exploit IgG uptake at low pH by surface receptors in attempts to understand the subsequent steps in IgG endocytosis. A handful of strategies were used. Some experiments were performed with adherent cells such as the Madin–Darby Canine Kidney cells wherein IgG transport was followed after uptake in pH6 medium [e.g., Ref. (2)]. More recently, both IgG and FcRn have been mutated to manifest high affinity for one another at physiological pH, so that uptake of IgG by the surface receptors of these cells can be studied at physiological pH without resorting to a low-pH uptake step [e.g., Ref. (3)]. In the neonatal rat, gut IgG trafficking has been followed after instillation of pH6 ligand into the gut lumen [e.g., Ref. (4)].

The last two decades have witnessed a spate of studies describing the intracellular trafficking that follows ligand uptake by surface receptors [14 papers from multiple laboratories in 18 years; see citations in Ref. (5)]. By ignoring alternative pathways, these studies appear to assume that this pathway is the major if not the only uptake pathway. They seem to ignore the possibility that this surface-receptor-initiated pathway may be different and distinct from a pathway that ensues after ligand binding to the receptor in the acidic endosome subsequent to non-specific pinocytosis of ligand. The latter pathway, first hypothesized by Brambell in his original formulation, has not yet been studied *in vitro* in isolated cells. Yet, this pathway is almost certainly the one utilized in the IgG degradation pathway and in the yolk sac transport pathway. And, there are solid reasons to believe that it is the pathway used for transport across the neonatal gut in mice and rats.

So, what accounts for ignoring this pathway for two decades, the pathway that begins with non-specific uptake of ligand and receptor recognition of ligand in acidic endosomes? Why has attention been directed solely at the pathway following ligand binding to surface-expressed receptor? The literature is not helpful in answering why a more direct approach has not been taken, why it has not been possible to learn how acidic endosomes take up IgG that has been non-specifically endocytosed and how FcRn then moves its ligand across the cell. Perhaps, it is enough for an essay of this sort, one person’s opinion, to point out that we workers in the field have passed over an important question, and the whole story of FcRn transport will not be readily understood unless we return to basic experiments and answer these fundamental questions. I invite my colleagues to respond to this challenge.

## Conflict of Interest Statement

The author declares that the research was conducted in the absence of any commercial or financial relationships that could be construed as a potential conflict of interest.
